# Risk Factors for Postoperative Complications among the Elderly after Plastic Surgery Procedures Performed under General Anesthesia

**DOI:** 10.1155/2018/7053839

**Published:** 2018-07-08

**Authors:** Kiyoko Fukui, Masaki Fujioka, Kazumi Yamasaki, Sho Yamakawa, Haruka Matsuo, Miho Noguchi

**Affiliations:** ^1^Department of Plastic and Reconstructive Surgery, National Hospital Organization Nagasaki Medical Center, Nagasaki, Japan; ^2^Department of Plastic and Reconstructive Surgery, Nagasaki University, Nagasaki, Japan; ^3^Department of Liver Internal Medicine, National Hospital Organization Nagasaki Medical Center, Nagasaki, Japan; ^4^Department of Clinical Research Center, National Hospital Organization Nagasaki Medical Center, Nagasaki, Japan

## Abstract

**Background:**

The frequency of surgery involving elderly patients has been increasing. The use of free tissue transfers in the elderly has been examined previously (Howard et al., 2005, Hwang et al., 2016, Grammatica et al., 2015, Serletti et al., 2000, and Sierakowski et al., 2017), whereas there have not been any such studies of plastic surgery procedures. We evaluated the risk factors for complications after plastic surgery procedures performed under general anesthesia in patients aged ≥75 years.

**Methods:**

The cases of patients aged ≥75 years who underwent plastic surgery procedures under general anesthesia at the Department of Plastic and Reconstructive Surgery, National Hospital Organization Nagasaki Medical Center, between 2009 and 2016 were reviewed retrospectively. Multiple logistic regression analysis was used to identify the risk factors for postoperative complications.

**Results:**

Two hundred and sixty-three cases were reviewed. Complications were seen in 137 patients. Age was not predictive of complications. The risk factors included a serum albumin level of <2.8 g/dl (odds ratio (OR): 2.96), an operative time of ≥120 min (OR: 6.22), and an American Society of Anesthesiologists performance status of ≥3 (OR: 2.39).

**Conclusions:**

Age is not contraindication for surgery in the elderly. It is important to assess comorbidities and perform surgical procedures as soon as possible to shorten the surgical period.

## 1. Introduction

The populations of developed countries have been aging progressively, and Japanese society is aging especially rapidly [1]. The feasibility of performing surgery in elderly patients has increased, but this has resulted in a greater frequency of postoperative complications because the functional capacity of such patients' organs is impaired [2]. Thus, it is important to clarify the risk factors for postoperative complications in elderly patients.

The complications of free flap reconstruction after oncological surgery in elderly patients have recently been discussed [2–6]. However, the risk factors for complications after plastic surgery procedures in the elderly have not been reported. The aim of this study is to evaluate the risk factors for postoperative complications after surgery performed under general anesthesia in patients aged over 75 years.

## 2. Materials and Methods

The cases of patients aged over 75 years who underwent plastic surgery procedures under general anesthesia at the Department of Plastic and Reconstructive Surgery, National Hospital Organization Nagasaki Medical Center, between 2009 and 2016 were evaluated retrospectively. Various preoperative and intraoperative variables were recorded, including age, gender, disease type, laboratory parameters (the levels of hemoglobin, albumin, and C-reactive protein and the white blood cell count), the subjects' preoperative ability to perform activities of daily living (ADL), the surgical procedure, the duration of general anesthesia, and the operative time. The subjects' preoperative ability to perform ADL was assessed based on two categories, i.e., whether or not the subjects maintained the ability to walk without support.

Comorbidity status was evaluated using the American Society of Anesthesiologists (ASA) performance status (PS) and the Charlson comorbidity index (CCI). The ASA PS is a scoring system that is used to define anesthesiological risk. The CCI is a grading system based on 16 medical conditions, and it is considered to be related to survival in hospitalized patients [3].

Postoperative complications and mortality were recorded, and overall complications were divided into two categories, i.e., into complications that were directly due to surgical procedures (surgical complications) and other complications (medical complications). Operative mortality was defined as deaths that occurred within 30 days of surgery.

All statistical analyses were performed using the SPSS 24.0 software. Multiple logistic regression analysis was used to assess the risk factors for postoperative complications, and p-values of <0.05 were regarded as statistically significant. Receiver operating characteristic (ROC) curves were used to estimate the optimal cut-off points for the serum albumin level and operative time.

## 3. Results

### 3.1. Patients

A total of 309 patients that were older than 75 years underwent surgery at our institution during the study period. Thirty-one patients were excluded because they underwent reconstruction after oncological surgery, including for laryngeal, oropharyngeal, or cervical esophageal cancer, and 15 patients were excluded because their preoperative laboratory data were insufficient. Thus, the cases of 263 patients were finally reviewed.

The subjects' median age at the time of surgery was 82 years (range: 75–99 years). There were 124 males (47.1%) and 139 females (52.9%). In total, 203 (77.2%) and 60 patients (22.8%) had ASA PS of ≤2 and ≥3, respectively. Moreover, 138 patients (52.3%) and 125 patients (47.5%) exhibited CCI scores of ≤2 and ≥3, respectively. The median operative time was 60 min (range: 11–451 min) (Table 1). In addition, 124 of 263 patients (47%) could not walk without assistance/a wheelchair or were bedridden before the operation.

### 3.2. Types of Disease

Thirty-five patients suffered peripheral arterial disease (PAD) with ulcers and/or necrosis; 47 patients had pressure ulcers; 19 patients had necrotizing fasciitis; and 31 patients developed ulcers caused by other diseases, such as diabetes, collagen disease, radiation, myelitis, or venous congestive disease. Twenty-six patients suffered traumatic injuries, such as facial fractures and hand injuries. Thirty-two patients suffered burns. Fifty-seven patients developed malignant tumors on their body surfaces. Two patients experienced scar contraction. Fourteen patients developed other conditions, such as blepharoptosis and facial nerve paralysis (Table 2).

### 3.3. Surgical Procedures

Forty-nine, 76, 59, and 3 patients underwent amputation, pedicled flap transfer, skin grafts, and free flap reconstruction, respectively. Twenty-six, 15, 20, and 15 patients underwent debridement, fracture repair, tumor resection, and other surgical procedures, respectively (Table 3). There were 133 surgical procedures in which the operative time was ≥60 min (pedicled flap transfer: 51, skin graft: 26, amputation: 22, fracture repair: 11, debridement: 6, free flap transfer: 3, and others: 14) (Table 4).

### 3.4. Complications

Overall complications were seen in 137 patients (52.1%). Surgical complications occurred in 80 patients (30.4%), and medical complications arose in 87 patients (33.1%) (Table 5).

The most common medical complication was delirium, which was seen in 73 patients. Pneumonia or atelectasis occurred in 9 patients, and 3 patients were affected by congestive heart failure, cardiac arrhythmia, or pulmonary edema. Other medical complications (renal failure, cholecystitis, and sepsis) occurred in 4 patients (Table 6).

Regarding surgical complications, 36 patients developed skin necrosis, 23 suffered skin graft loss, 11 developed hematomas/seromas, 9 contracted infections, and 1 developed lymphorrhea (Table 7).

Surgical and medical complications occurred in 54 and 55 patients, respectively, who underwent procedures longer than ≥60 min. Among the surgical procedures that took ≥60 min, pedicled flap transfers for pressure ulcers (26 patients) caused the most complications, followed by skin grafts (12 patients) and then amputations (7 patients).

Six patients died after surgery (2.28%). Three patients died of pneumonia, 2 patients died of sepsis (1 patient suffered necrotizing fasciitis caused by a group G beta-hemolytic streptococcus, and another patient developed sepsis caused by necrosis of the small bowel), and 1 patient died due to an advanced malignant fibrous histiocytoma.

The frequency of each type of complication is shown according to the international Clavien-Dindo classification in Table 8.

### 3.5. Risk Factors for Postoperative Complications

#### 3.5.1. Overall Complications

Age, gender, the preoperative levels of hemoglobin and C-reactive protein, the preoperative white blood cell count, the surgical procedure, the duration of general anesthesia, and the CCI score were not predictive of the overall frequency of overall complications. Conversely, the preoperative serum albumin level, the operative time, and the ASA PS were found to be associated with the overall frequency of overall complications (Table 9). Specifically, a serum albumin level of <2.8 g/dl (odds ratio [OR]: 2.96 [95% confidence interval [CI]: 1.68–5.19]; p < 0.001), an operative time of ≥60 min and <120 min (OR: 1.95 [95% CI: 1.09–3.50]; p = 0.03), an operative time of ≥120 min (OR: 6.22 [95% CI: 2.71–14.26]; p < 0.001), and an ASA PS of ≥3 (OR: 2.39 [95% CI: 1.24–4.63]; p < 0.001) were identified as risk factors for overall complications.

#### 3.5.2. Medical Complications

The preoperative serum albumin level, operative time, and the subjects' preoperative ability to perform ADL were found to be associated with medical complications (Table 10). Specifically, the risk factors for medical complications were shown to include a serum albumin level of <2.8 g/dl (OR: 2.49 [95% CI: 1.36–4.55]; p < 0.001), an operative time of ≥62 min (OR: 1.92 [95% CI: 1.10–3.33]; p = 0.02), and the subjects' preoperative ability to perform ADL (patients who cannot walk without assistance/a wheelchair or were bedridden (OR: 2.03 [95% CI: 1.10–3.72]; p = 0.02)).

#### 3.5.3. Surgical Complications

The operative time and type of disease were found to be associated with surgical complications (Table 11). Specifically, the risk factors for surgical complications included an operative time of ≥62 min and ulcers, including PAD, pressure ulcers, necrotizing fasciitis, and other ulcers.

## 4. Discussion

The proportion of the elderly has been increasing in Japan. The Japan Geriatrics Society defines 65- to 74-year-old people as “pre old”, 75- to 89-year-old people as “old”, and people aged over 90 years as “oldest-old” [7]. In total, 12.5% of the Japanese population is over 75 years old [1]. Chronic ulcers caused by PAD and pressure ulcers are particularly common in elderly people [8], and these wounds are associated with discharges, infection, foul smells, and severe ischemic pain and require frequent dressing changes. In order to improve the quality of life of patients with such conditions, it is expected that the frequency of surgery involving elderly patients will increase. Thus, it is important to evaluate the risk factors for postoperative complications in the elderly after surgery performed under general anesthesia.

Recently, the use of free tissue transfers after oncological surgery in elderly patients has been examined [2–6], and a relationship was reported to exist between age and postoperative complications. Hwang et al. reviewed 41 studies about complications in the elderly after free flap surgery and reported that the overall and surgical complications rates increased with age [2]. On the other hand, Howard et al. reported that the frequency of medical complications was increased in the over 80 years age group, but not the 70 to 79 years age group; however, no age-related increase in the frequency of surgical complications was detected [3]. Grammatica et al. reported that microvascular reconstruction resulted in medical complications more frequently in the elderly, but the frequency of surgical complications was not affected by age [4]. Therefore, we considered that it would be useful to study the risk factors for postoperative complications in elderly patients who underwent surgery performed under general anesthesia. In our study of plastic surgery procedures, neither the overall frequency of complications nor the frequencies of medical or surgical complications increased with age.

As for other risk factors for postoperative complications, Serletti et al. reported that in microvascular surgery patients with higher ASA PS suffered medical complications more frequently, but the frequency of surgical complications was not increased in this group. Furthermore, a longer operative time was found to be associated with an increased frequency of surgical complications [5]. On the other hand, Howard et al. reported that alcohol consumption and coronary artery disease are risk factors for medical and surgical complications [3]. In our study of plastic surgery procedures, a longer operative time was associated with an increased frequency of complications, including both medical and surgical complications, and it appeared that the operative time had the strongest influence on the risk of postoperative complications. Of the procedures that took longer than 60 min, many involved pedicled flap transfers for pressure ulcers, skin graft transfers for ulcers or burn wounds, or major amputation for PAD (Table 4). It would be difficult to shorten the operating times of such procedures. However, improving the skill of the operator or treating patients with negative pressure wound therapy to cause their wounds to contract before they are resurfaced might help to shorten the operating time of such procedures to <60 min. In addition, a lower serum albumin level was demonstrated to be associated with increased frequencies of both overall and medical complications. The patients with ulcers (pressure ulcers, PAD, necrotizing fasciitis, or other ulcers) developed postoperative complications more frequently than the other patients, and these patients were also malnourished. In each of these cases, we consulted the nutritional support team and attempted to alleviate the patient's malnutrition.

Moreover, a higher ASA PS was shown to be associated with an increased frequency of overall complications. Concerning the patients' ability to perform preoperative ADL, patients who needed physical assistance suffered medical complications more frequently. It appeared that patients' preoperative medical status, for example, their nutritional status and their ability to perform ADL, had a greater influence on their risk of postoperative complications than age.

As for the patients' preoperative and postoperative functional status, 124 of 263 patients (47%) could not walk without assistance/a wheelchair or were bedridden before the operation. For example, some patients suffered from PAD or pressure ulcers, and many were bedridden or confined to a wheelchair before the operation. Therefore, their ability to perform ADL did not change very much after surgery because they were already bedridden before the operation. The remaining 139 patients (53%) were able to move independently before the operation, and 104 patients were suffering from conditions other than ulcers (facial fractures, hand injuries, malignant tumors, etc.). These patients were discharged shortly after the operation, and so their ability to perform ADL did not change very much.

The patients suffering from ulcers, including pressure ulcers and PAD, experienced postoperative surgical complications more frequently than the other patients. Bamba et al. reported that the surgical complications rate after pressure ulcer reconstruction was 58.7% (162 out of 276 patients) [9]. Keys et al. reported that 110 out of 231 flap transfer procedures (49%) for pressure ulcers resulted in postoperative dehiscence [10]. Regarding PAD, Beaulieu et al. found that readmission (because of infection, nonhealing wounds, or ischemia) was necessary in 13.9% of patients that underwent minor amputations for PAD [11]. Curran et al. reported that the complications rate was 43% (among 5732 patients) after amputation for PAD [12]. In our study, surgical complications occurred in 80 patients (30.4%), which is a similar frequency to those reported previously.

In conclusion, age is not a contraindication for plastic surgery procedures performed under general anesthesia in the elderly. In cases involving elderly patients, it is important to assess preoperative medical conditions and comorbidities carefully and to perform surgical procedures as soon as possible in order to shorten the surgical period.

## Figures and Tables

**Table 1:** Baseline characteristics of all patients.

All patients	*n* = 263
Age (years)	
Median	82
Range	75–99

Males, *n* (%)	124 (47.1)
Females, *n* (%)	139 (52.9)

ASA PS	
≤2, *n* (%)	203 (77.2)
≥3, *n* (%)	60 (22.8)

CCI	
≤2, *n* (%)	138 (52.3)
≥3, *n* (%)	125 (47.5)

Operative time (min)	
Median	60
Range	11–451

**Table 2:** Types of disease.

Conditions	No. of patients
Peripheral arterial disease	35
Pressure ulcers	47
Necrotizing soft tissue infections	19
Other ulcers	31
Traumatic injuries	26
Burns	32
Tumors	57
Scars	2
Others	14

Total	263

**Table 3:** Surgical procedures.

Surgery	No. of patients
Amputation	49
Pedicled flap transfer	76
Skin graft	59
Free flap transfer	3
Debridement	26
Fracture repair	15
Tumor resection	20
Others	15

Total	263

**Table 4:** Surgical procedures involving operative times of ≥60 min.

Surgery	No. of patients
Pedicled flap transfer	51
Skin graft	26
Amputation	22
Fracture repair	11
Debridement	6
Free flap transfer	3
Others	14
Total	133

**Table 5:** Complications.

Complications	No. of patients
Overall	137 (52.1%)
Medical	87 (33.1%)
Surgical	80 (30.4%)

**Table 6:** Medical complications.

Medical complications	No. of patients
Delirium	73
Pneumonia, atelectasis	9
CHF^*∗*^, arrhythmia, pulmonary edema	3
Others	4

CHF: congestive heart failure.

**Table 7:** Surgical complications.

Surgical complications	No. of patients
Skin necrosis	36
Loss of skin graft	23
Hematomas/seromas	11
Infection	9
Lymphorrhea	1

**Table 8:** Frequencies of medical and surgical complications according to the Clavien-Dindo classification.

Complication type	Clavien-Dindo grade						
	I	II	IIIa	IIIb	IVa	IVb	V
Medical	3	78	1	0	5	0	5
Surgical	19	11	36	13	0	0	1

**Table 9:** Overall complications.

Variables	OR	95% CI	p-values
Alb (g/dl)			
≥2.8	1 (ref)		
<2.8	2.96	1.68–5.19	0.00

Operative time (min)			
<60	1 (ref)		
≥60, <120	1.95	1.09–3.50	0.03
≥120	6.22	2.71–14.26	0.00

ASA PS			
≤2	1 (ref)		
≥3	2.39	1.24–4.63	0.01

**Table 10:** Medical complications.

Variables	OR	95% CI	p-values
Alb (g/dl)			
≥2.8	1 (ref)		
<2.8	2.49	1.36–4.55	0.00

Operative time (min)			
<62	1 (ref)		
≥62	1.92	1.10–3.33	0.02

Preoperative ADL			
Walk without support	1 (ref)		
Physical assistance^*∗*^	2.03	1.10–3.72	0.02

^*∗*^Physical assistance: patients who cannot walk without assistance/a wheelchair or were bedridden.

**Table 11:** Surgical complications.

Variables	OR	95% CI	p-values
Operative time (min)			
<62	1 (ref)		
≥62	3.20	1.83–5.61	0.00

Types of disease			
Ulcers^*∗*^	1 (ref)		
Others^*∗∗*^	0.45	0.25–0.78	0.01

^*∗*^Ulcers: PAD, pressure ulcers, necrotizing fasciitis, other ulcers (those associated with diabetes, collagen, radiation, etc.)

^*∗∗*^Others: traumatic injuries, tumors, or scars.
